# First case report of *Shewanella indica* isolated from a hospitalized patient in Serbia

**DOI:** 10.3389/fmed.2025.1715579

**Published:** 2026-01-06

**Authors:** Brankica Filipić, Vladimir Žugić, Majda Golob, Martina Hrast Rambaher, Lidija Bošković, Marina T. Milenković

**Affiliations:** 1 Department of Microbiology and Immunology, Faculty of Pharmacy, University of Belgrade, Belgrade, Serbia; 2 Clinical Hospital Center Zvezdara, University of Belgrade, Belgrade, Serbia; 3 Veterinary Faculty, Institute of Microbiology and Parasitology, University of Ljubljana, Ljubljana, Slovenia; 4 Department of Pharmaceutical Chemistry, Faculty of Pharmacy, University of Ljubljana, Ljubljana, Slovenia

**Keywords:** broth microdilution, case report, genome sequencing, *Shewanella indica*, species identification

## Abstract

Over the past decade, *Shewanella* spp. have been increasingly recognized as opportunistic pathogens, particularly in patients with malignancies, neutropenia, severe heart failure, renal insufficiency, and hepatobiliary diseases. *Shewanella indica* is a rarely reported species within the *Shewanella* genus, and its role in human infection remains poorly documented. In this study, we report the first case of *S. indica* isolated from the stool of a critically ill 72-year-old man in Serbia with multiple pre-existing comorbidities and co-infections. Initial identification using the VITEK 2 system misclassified the strain as *Shewanella algae*, while species-level identification by the matrix-assisted laser desorption/ionization-time-of-flight mass spectrometry (MALDI-TOF MS) was inconclusive. However, whole-genome sequencing (WGS) definitively identified the isolate as *S. indica* and revealed a multidrug-resistant profile together with numerous virulence-associated genes. Minimum inhibitory concentrations (MICs) were determined for 32 antibiotics, although interpretation was constrained by the lack of species-specific breakpoints. This case highlights the diagnostic challenges in differentiating *Shewanella indica*, discusses its possible clinical roles, and underscores the value of genomic tools for accurate identification. It also reinforces the importance of recognizing rare pathogens in complex clinical scenarios.

## 1 Introduction

The genus *Shewanella* comprises Gram-negative, rod-shaped, motile bacteria. Members of this genus are facultative anaerobic, oxidase-positive, and catalase-positive. Some species can ferment carbohydrates such as D-glucose and produce H_2_S. They can degrade gelatine and reduce nitrate into nitrite. The majority of clinically relevant *Shewanella* strains also possess DNase, protease, and gelatinase activity, which may contribute to pathogenicity (1). According to the GenBank data, more than 100 species have been identified to date. These bacteria occur naturally in marine environments, particularly in warmer geographical climates. However, owing to their physiological and respiratory versatility, *Shewanella* spp. can adapt to a broad range of ecological niches (1, 2). In the last decade, several species have emerged as opportunistic human pathogens. *Shewanella* infections are most often reported in immunocompromised patients with malignancies, neutropenia, advanced heart failure, renal insufficiency, and hepatobiliary disease (3–5). However, infections in previously healthy individuals have also been described. Among the most clinically relevant species are *Shewanella algae*, *Shewanella putrefaciens*, and *Shewanella xiamenensis* (2, 3). The most common clinical manifestations of *Shewanella* infection are soft-tissue infections (including cellulitis, abscess, and necrotizing fasciitis). However, invasive diseases such as bloodstream infections (bacteremia and septicemia) and intra-abdominal infections have also been documented (2, 4, 6). Over the past decade, members of this genus have attracted increasing attention from the medical community due to their ability to develop resistance to multiple antibiotic classes, including beta-lactams, aminoglycosides, quinolones, third- and fourth-generation cephalosporins, and carbapenems (7–9).

In the present study, we report the first case of *Shewanella indica* isolated from the stool of a hospitalized patient with multiple and complex underlying diseases. The isolate was initially identified as *S. algae* by the VITEK 2 system; however, whole-genome sequencing using the Illumina HiSeq platform finally confirmed it as *Shewanella indica.* Based on a brief search of the available literature, and to the best of our knowledge, no previous cases of *Shewanella* infection have been documented in Serbia or in the wider Balkan region. This case therefore represents the first reported instance of a ‘probable’ *S. indica* infection in this area, highlighting both the challenges of accurate species-level identification and the clinical relevance of these rare pathogens. In addition, the study provides whole-genome sequencing and analysis of the strain and reports MIC values for 32 antibiotics. It serves as a reminder for clinicians and microbiologists to consider *S. indica* as a potential emerging pathogen and underscores the importance of accurate species identification, particularly the correct differentiation between *S. indica* and *S. algae*.

## 2 Case description

A 72-year-old man with symptoms of pneumonia was admitted to the Department of Pulmonology at the Clinical Hospital Centre Zvezdara in Belgrade, Serbia. One month earlier, the patient had been hospitalized at a psychiatric hospital (2–30 July 2024) for alcohol withdrawal therapy, during which he was treated with antibiotics for a suspected urinary tract infection and received intensive diuretic therapy for heart failure. However, his general condition deteriorated, and he experienced significant blood oxygen desaturation, with levels falling below 70%. This critical decline prompted his transfer to the Clinical Department of Pulmonology for further evaluation and treatment.

Clinical examination on admission showed a conscious and awake but uncommunicative patient with bradykinesia and dyspnea. He was afebrile, without osteomuscular anomaly, cachectic, and dehydrated, with diffuse hematomas and ongoing diarrhea, providing the overall impression of a severely ill patient. A multidisciplinary evaluation was performed involving a cardiologist, a pulmonologist, a neurologist, and a surgeon. Diagnostic imaging included a cranial CT scan, which showed no acute lesions; abdominal ultrasound revealed diffuse colonic edema and mild, non-significant ascites. Auscultatory attenuated diffuse breath sounds in the lungs and chest X-rays indicated signs of right-sided pneumonia. Cardiac examination showed an irregular rhythm, consistent with atrial fibrillation, with clear heart sounds and no murmurs. Heart rate was 80 beats/min, and blood pressure was 80/60 mmHg. The abdomen was noted to be soft, painfully insensitive, and without organomegaly. On admission, an electrocardiogram (ECG) showed atrial fibrillation, with a ventricular rate of 70 beats/min. Laboratory tests revealed leukocytosis with anemia, azotemia, hypoalbuminemia, and elevated D-dimer levels. The viral markers (human immunodeficiency virus (HIV), hepatitis C virus (HCV), and hepatitis B virus (HBV)) and blood cultures were negative. Urine sediment suggested infection, although the urine culture was negative. Methicillin-resistant *Staphylococcus aureus* (MRSA) was isolated from both the nasal swab and bronchial aspirate. A throat swab revealed heavy growth of *Candida* spp. Stool examination for *Clostridium difficile* was positive, and *Shewanella* spp. was incidentally isolated from the stool. *C. difficile* was identified using the Glutamate Dehydrogenase (GDH) test, followed by an immunochromatographic test for toxin detection. *Shewanella* spp. was isolated in pure culture on Salmonella Shigella (SS) agar. For the *Shewanella* spp., there were no data available for seafood intake, fish handling, wounds, travel, seawater contact, or household exposures. Arterial blood gas analysis initially indicated metabolic acidosis, progressing to mixed respiratory/metabolic acidosis with lethal hypercapnia. This necessitated endotracheal intubation and initiation of mechanical ventilation (MV). Bronchoscopy was performed through an orotracheal tube, whereby a large amount of mucopurulent content was aspirated. During hospitalization, the patient was treated intravenously (IV) with meropenem (IV 3 × 1 g), ciprofloxacin (IV 2 × 100 mg), antifungal agents, bronchodilators, low-molecular-weight heparin (LMWH), proton-pump inhibitors (PPIs), antiarrhythmics, inotropic therapy, and diuretics. In addition, he received albumin replacement and red blood cell transfusion and controlled oxygen therapy with MV support.

From the very beginning, the patient was in a critical condition. Within hours of admission, he experienced progressive respiratory deterioration and was transferred to the Respiratory Intensive Care Unit (ICU). He was intubated and placed on MV under continuous monitoring of vital parameters.

An anesthesiologist was consulted daily to adjust and optimize ventilation parameters. On 4 August, the patient was extubated and further treated with an oxygen mask, and his blood pressure remained stable. However, in the following days, progressive oligoanuria, azotemia, hyperkalemia, and worsening metabolic acidosis developed. On the morning of 7 August, the patient experienced sudden and severe deterioration in his general condition. He was found without spontaneous cardiac activity and respiration. Cardiopulmonary resuscitation (CPR) was initiated but proved unsuccessful. *Exitus letalis* was subsequently declared (Table 1).

**Table 1:** Chronological overview of clinical events and interventions.

Date	Event/intervention	Findings/notes
July 2–30, 2024	Hospitalization in a psychiatric facility	Treated for alcohol withdrawal; suspected UTI (antibiotics), and heart failure (diuretics)
Late July 2024	Clinical deterioration	Blood oxygen desaturation <70%, prompting transfer
Late July 2024	Admission to the Pulmonology Department (Clinical Hospital Center Zvezdara)	Awake, aware, but uncommunicative. Dyspnea, cachexia, dehydration, and diffuse hematomas
	Initial diagnostics	Cranial CT: no acute lesions; abdominal US: colonic edema and mild ascites
	Chest X-ray	Signs of right-sided pneumonia
	ECG	Atrial fibrillation (ventricular rate: 70/min)
	Vitals	BP 80/60 mmHg, HR 80/min
	Laboratory findings	Leukocytosis, anemia, azotemia, hypoalbuminemia, ↑ and D-dimer
	Microbiology	MRSA (nasal + bronchial), *Candida* spp. (throat), *C. difficile* (stool), and *Shewanella* spp. (stool)
	Arterial blood gas	Mixed respiratory/metabolic acidosis → lethal hypercapnia
~Late July 2024	Endotracheal intubation and mechanical ventilation (MV)	Due to respiratory failure
	Bronchoscopy	Purulent secretions
	Specialist consults	Cardiologist, gastroenterologist, and nephrologist
	Treatments started	Antibiotics, antifungals, desobstructive therapy, LMWH, PPIs, inotropes, and diuretics
	Transfer to the ICU	With MV, inotropic support, and daily anesthesiologist input
4 August, 2024	Extubated, switched to an oxygen mask	BP stable post-extubation
5–6 August, 2024	Oligoanuria, worsening azotemia, and ↑ potassium	Metabolic acidosis returns
7 August, 2024	Sudden cardiac arrest, unsuccessful CPR	*Exitus letalis*

## 3 Bacteriological examinations and antimicrobial susceptibility testing

**Figure 1:**
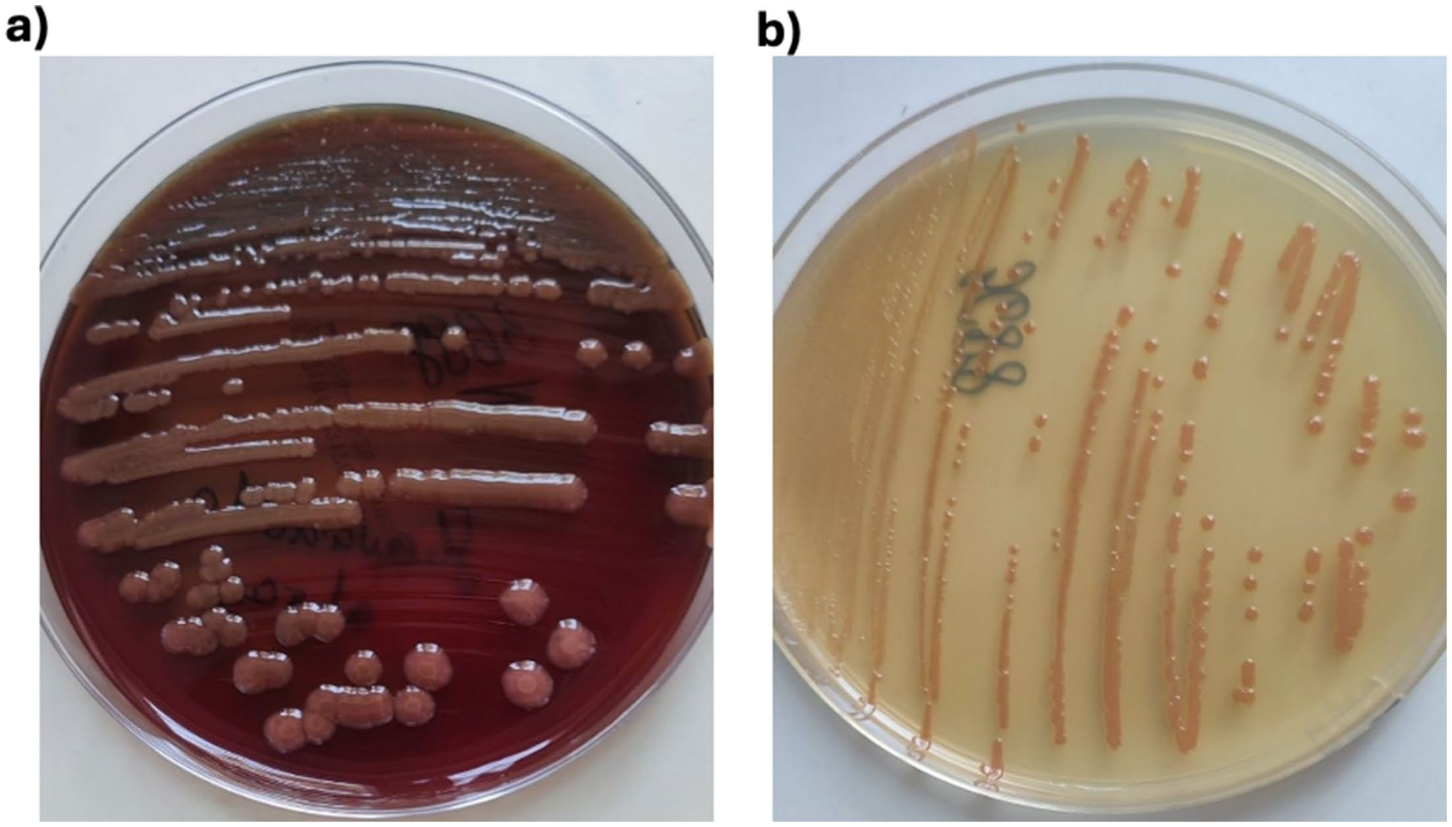
Colony morphology of *Shewanella indica* on **(a)** blood agar and **(b)** HiCrome™ UTI agar.

Given the absence of previously published cases of isolated *Shewanella* spp. in Serbia, a comprehensive microbiological and genomic investigation was undertaken following the patient’s death to identify and characterize the isolated pathogen. Primary culture of *Shewanella* spp. from the stool sample was isolated on SS agar (Salmonella Shigella agar, HiMedia, India) and Xylose Lysine Deoxycholate agar (XLD agar, HiMedia, India). After 24 h of incubation at 35 °C under ambient conditions, the suspected colonies from the SS agar were subcultured onto Tryptic Soy Agar, blood agar, and HiCrome™ UTI agar (used in the laboratory as a part of routine panel media). The colonies on blood agar were non-hemolytic, circular (2–5 mm in diameter), and smooth, ranging in color from dark pink to brown. On selective chromogenic media, there are no standardized color reactions for *Shewanella* because these media are designed for *Enterobacterales* or specific pathogens. The colony morphology of *Shewanella* spp. on blood agar and HiCrome™ UTI agar is shown in Figure 1.

Presumptive colonies were subjected to the VITEK 2 System (bioMérieux, France), which identified the strain as *S. algae.* MALDI-TOF MS (Bruker Daltonics, Germany; MBT Compass HT software and library version 5.1.220) analysis yielded the highest similarity score to *S. indica* (log score 2.31) and *S. algae* (log score 2.20), but species-level resolution remained inconclusive. Thus, accurate identification of the strain was not achievable using VITEK 2 or MALDI-TOF MS alone.

To accurately identify *Shewanella* spp., whole-genome sequencing was performed using the Illumina HiSeq platform. The resulting sequence was deposited in the National Center for Biotechnology Information (NCBI) database under accession number JBLYUA000000000 (number of contigs: 149; total length: 4,458,760 bp; GC: 52.25; genome coverage: 12.0x).

Genome analysis confirmed the isolate as *S. indica*. Multilocus sequence typing (MLST; genomicepidemiology.org) indicated closest similarity to sequence type (ST) 41, although this ST has not been characterized in scientific publications. This finding suggests that the isolate may represent a local clinical strain identified within a Serbian hospital setting.

Antibiotic resistance genes (ARGs) were identified using ResFinder 4.7.2 with a threshold identity of 98% and a selected minimum length of 60% (10), revealing determinants conferring resistance to tobramycin, amikacin, beta-lactams, tetracyclines, and sulfamethoxazole. The results obtained using ResFinder 4.7.2 are shown in Table 2, Supplementary Table S1. According to the study by Araújo et al. (11), carriage of the *qnrA2* gene is a characteristic feature of *S. indica*, and it is more frequently detected in *S. indica* than in *S. algae*. Genome analysis of the *S. indica* isolate described in this study, performed using ResFinder, confirmed the presence of the *qnrA2* gene with 100% sequence identity (position of detected gene—Node 1, contig position 143,203–143,859; Supplementary Table S1). Furthermore, basic local alignment search tool (BLAST) analysis^1^ comparing the genome sequence of the isolate described in this study with the *qnrA2* gene from *S. indica* strain Sh16 (accession number OK173611) also revealed 100% identity of *qnrA2* gene sequences (*E*-value: 0.0).

**Table 2:** Identification of acquired genome resistance genes using ResFinder.

Antimicrobial	Class	WGS-predicted phenotype	Genetic background	Position in contig
Tobramycin	Aminoglycoside	Resistant	aac(6′)-Ib-Hangzhou (aac(6′)-Ib-Hangzhou_FJ503047)	NODE_14;48,908…49,426
Amikacin	Aminoglycoside	Resistant	aac(6′)-Ib-Hangzhou (aac(6′)-Ib-Hangzhou_FJ503047)	NODE_14;48,908…49,426
Ciprofloxacin	Quinolone	Resistant	qnrA2 (qnrA2_AY675584)	NODE_1; 143,203…143,859
Amoxicillin	Beta-lactam	Resistant	blaOXA-2 (blaOXA-2_DQ112222)	NODE_14; 49,514…50,341
Amoxicillin + clavulanic acid	Beta-lactam	Resistant	blaOXA-2 (blaOXA-2_DQ112222)	NODE_14; 49,514…50,341
Ampicillin	Beta-lactam	Resistant	blaOXA-2 (blaOXA-2_DQ112222)	NODE_14; 49,514…50,341
Ampicillin + clavulanic acid	Beta-lactam	Resistant	blaOXA-2 (blaOXA-2_DQ112222)	NODE_14; 49,514…50,341
Ceftazidime	Beta-lactam	Resistant	blaOXA-2 (blaOXA-2_DQ112222)	NODE_14; 49,514…50,341
Piperacillin	Beta-lactam	Resistant	blaOXA-2 (blaOXA-2_DQ112222)	NODE_14; 49,514…50,341
Unknown beta-lactam	Beta-lactam	Resistant	blaOXA-SHE (blaOXA-SHE_AY066004)	NODE_5; 92,818…93,687
Sulfamethoxazole	Folate pathway antagonist	Resistant	sul1 (sul1_U12338)	NODE_14; 47,559…48,398
Erythromycin	Macrolide	Resistant	mph(A) (mph(A)_D16251)	NODE_14; 41,343…42,248
Azithromycin	Macrolide	Resistant	mph(A) (mph(A)_D16251)	NODE_14; 41,343…42,248
Spiramycin	Macrolide	Resistant	mph(A) (mph(A)_D16251)	NODE_14; 41,343…42,248
Telithromycin	Macrolide	Resistant	mph(A) (mph(A)_D16251)	NODE_14; 41,343…42,248
Tetracycline	Tetracycline	Resistant	tet(A) (tet(A)_AJ517790)	NODE_14; 31,229…32,428

To identify potential plasmid-associated sequences, the genome was analyzed using PlasmidFinder 2.1,^2^ which did not detect any plasmids in the isolated *Shewanella* strain. Additionally, based on the study by Cerbino et al. (12), a comparative analysis was conducted using BLASTX and BLASTP.^3^ The genome was compared against plasmid sequences previously identified in various *Shewanella* species (accession numbers: AAN52940.1, ALI93255.1, ASK71597.1, PKI04909.1, AEH16402.1, ABK50444.1, ASK71507.1, WP_011840021.1, and ACM47539.1) (12), and no significant similarity to known plasmid sequences was observed.

Screening with the Virulence Factor Database [VFDB; (13)] revealed a broad range of virulence-associated genes linked to adherence, antiphagocytosis, chemotaxis and motility, iron acquisition, quorum sensing, colonization, and immune evasion (Supplementary Table S2). Additionally, as part of the virulence factor detection, we examined the presence of collagenase in the isolated strain, since a previous study has shown that collagenase is present in the genome of *S. algae* but cannot be detected in the genome of *S. indica* (14). We identified a collagenase sequence from *Shewanella* spp., designated as UPI001EBA764A, and this sequence corresponds to a putative collagenase-like protein from a *Shewanella* species annotated in the UniParc database. Comparison of this sequence with the genome of the *S. indica* isolate described in our case report did not reveal the presence of collagenase. Furthermore, a search of the annotated *S. indica* genome confirmed the absence of collagenase, and analysis using the Virulence Factor Database (VFDB) did not identify any metalloproteinases (Supplementary Table S2).

**Figure 2:**
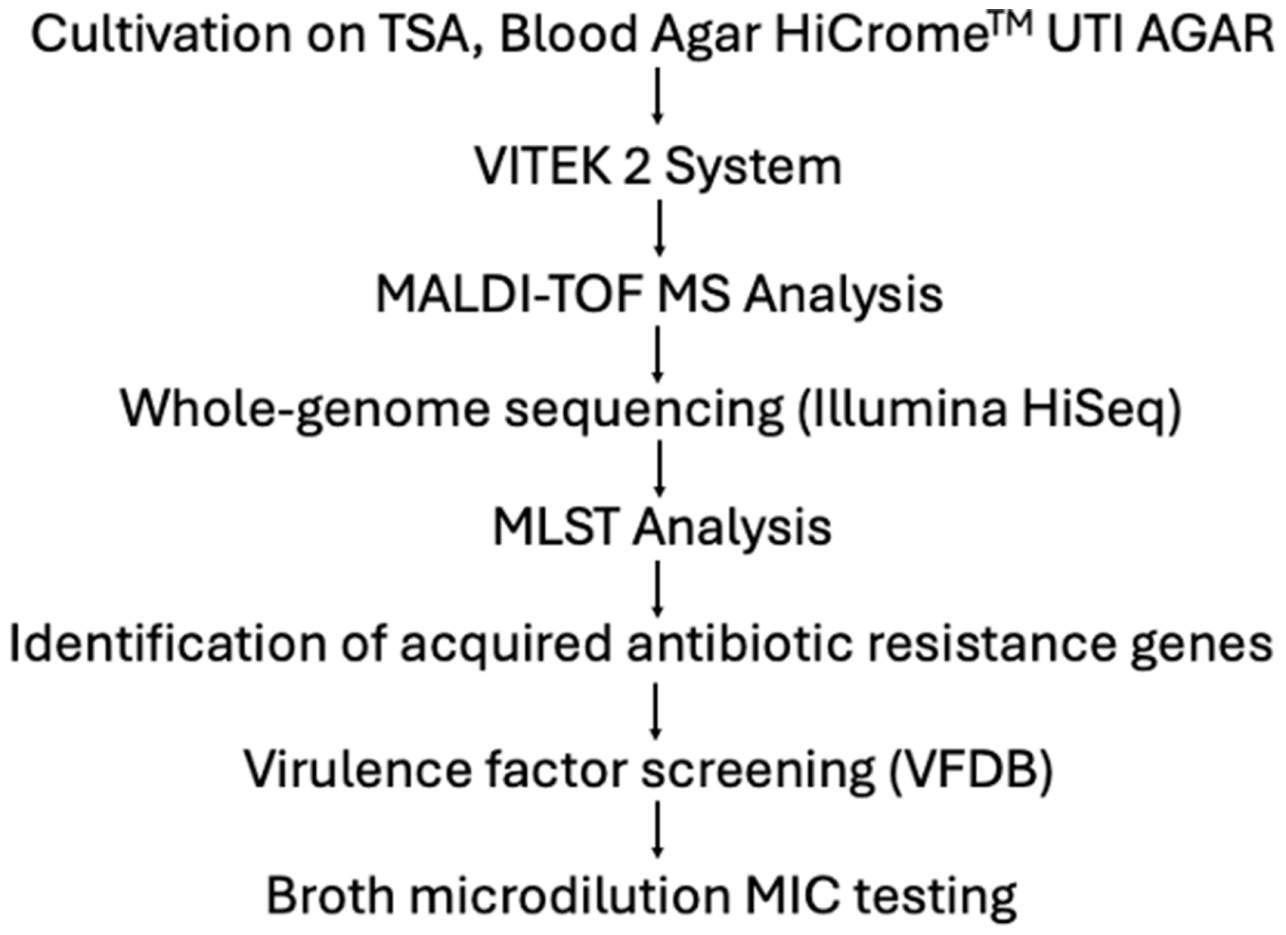
Chronological overview of the microbiological and genomic investigation of the *Shewanella indica* isolate.

To visually summarize the procedures and methods described in the case report, Figure 2 presents a comprehensive chronological overview of the microbiological and genomic investigations performed on the *Shewanella indica* isolate, highlighting key methodologies.

To assess antimicrobial resistance, broth microdilution testing was performed using three commercial 96-well plates (Sensititre GN3F, EUVSEC3, and EUVSEC2; Sensititre, Trek Diagnostic Systems, Thermo Scientific, East Grinstead, UK). *S. indica* was phenotypically tested for susceptibility to 32 different antimicrobials: amikacin (AMI), ampicillin (AMP), ampicillin/sulbactam (A/S), azithromycin (AZI), aztreonam (AZT), cefazolin (FAZ), cefepime (FEP), cefpodoxime (POD), cefotaxime (FOT), cefotaxime with clavulanic acid (F/C), cefoxitin (FOX), ceftazidime (TAZ), ceftazidime with clavulanic acid (T/C), ceftriaxone (AXO), cefuroxime (FUR), chloramphenicol (CHL), ciprofloxacin (CIP), colistin (COL), ertapenem (ETP), gentamicin (GEN), imipenem (IMI), meropenem (MERO), nalidixic acid (NAL), piperacillin/tazobactam constant 4 (P/T4), sulfamethoxazole (SMX), temocillin (TRM), tetracycline (TET), ticarcillin/ clavulanic acid constant 2 (TIM2), tigecycline (TGC), tobramycin (TOB), trimethoprim (TMP), and trimethoprim/sulfamethoxazole (SXT). MIC values were determined after 20 h of incubation at 35 °C under aerobic conditions. According to the European Committee on Antimicrobial Susceptibility Testing (EUCAST) and Clinical and Laboratory Standards Institute (CLSI) standards, the MIC endpoint was defined as the lowest concentration of the antimicrobial agent that inhibited visible bacterial growth compared with the positive growth control. For trimethoprim and trimethoprim/sulfamethoxazole, the MIC was read as the lowest concentration that inhibited at least 80% of growth relative to the control. Interpretation was limited by the lack of species-specific clinical breakpoints for *Shewanella* in EUCAST and CLSI guidelines. In line with previous studies involving *Shewanella* species, MIC interpretation was performed using the CLSI M100 34th edition, Table 2B–5 (2024), applying breakpoints designated for “Other Non-*Enterobacterales*” (Table 3, the antibiotics are listed in alphabetical order) (15). As MIC values for certain tested antibiotics could not be interpreted even after using CLSI guidelines, the obtained results were included to serve as a reference for future research related to *S. indica.*

**Table 3:** Minimum inhibitory concentration (MIC) values for the tested antibiotics.

Antibiotic	MIC (μg/mL)	CLSI, 2024
Amikacin	≤8	S
Ampicillin	>32	Ν.Α.
Ampicillin/sulbactam	≤ 4/2	Ν.Α.
Azithromycin	4	Ν.Α.
Aztreonam	>32	R
Cefazolin	>32	*Ν*.*Α*.
Cefepime	16	I
Cefotaxime	16	I
Cefotaxime/clavulanic acid	0.5/4	Ν.Α.
Cefoxitin	16	Ν.Α.
Cefpodoxime	8	Ν.Α.
Ceftazidime	128	R
Ceftriaxone	4	S
Cefuroxime	>32	Ν.Α.
Cephalothin	>16	Ν.Α.
Chloramphenicol	≤8	Ν.Α.
Ciprofloxacin	>4	R
Colistin	≤1	Ν.Α.
Ertapenem	4	Ν.Α.
Gentamicin	≤2	S
Imipenem	0.5	S
Meropenem	≤1	S
Nalidixic acid	>64	Ν.Α.
Piperacillin/tazobactam	≤16/4	S
Sulfamethoxazole	512	R
Temocillin	16	Ν.Α.
Tetracycline	32	R
Ticarcillin/clavulanic acid	64/2	Ν.Α.
Tigecycline	≤1	Ν.Α.
Tobramycin	≤4	S
Trimethoprim	4	Ν.Α.
Trimethoprim/sulfamethoxazole	≤0.5/9.5	S

The interpretation (S/I/R) is based on CLSI M100 (2024) breakpoints for “Other Non-*Enterobacterales*.” MIC values were determined after 20 h of incubation at 35 °C under aerobic conditions.

## 4 Discussion

*Shewanella indica*, described in this case report, was first isolated from marine sediment in India (16). Subsequently, Li et al. (17) reported its isolation from an intact skin abscess of a stranded Bryde’s whale (*Balaenoptera edeni*) in the northern Beibu Gulf, China, identified through a combination of biochemical tests and 16S rRNA sequencing (17). In the same year, Tamez et al. (14) characterized putative virulence factors in the genome of *S. indica* BW, which was also isolated from the Bryde’s whale. This study also provided further insights into its pathogenic potential (14).

Despite these findings, associations between *S. indica* and human disease remain poorly documented in the literature. A recent study from China identified *S. indica* in stool samples (*n* = 5) from patients with diarrhea (18). Following a comprehensive review of the literature, no cases of *S. indica* isolated from human clinical samples have been reported in Serbia or the broader Balkan region to date.

In the present case report, we describe the first Serbian strain of *S. indica*, isolated from a 72-year-old man with a ‘probable’ *S. indica* infection. The infection caused by the genus *Shewanella* is complex and could be due to a variety of factors. Considering the fact that the blood culture was negative and the patient had a low standard of living and poor hygiene habits, we can assume that the oral ingestion of the microorganism was the route of ‘probable’ infection. The clinical relevance of this finding is challenging to assess, as *S. indica* was co-isolated with another pathogen from the patient’s stool sample. The patient had multiple comorbidities, including pneumonia, heart failure, and concurrent infections with MRSA and *Clostridium difficile*, all of which likely contributed to his rapid clinical deterioration and unfavorable outcomes. Previous studies have shown that most reported *Shewanella* infections occur in older adults (over 60 years of age), with a male predominance. Yu et al. (2) reported a male-to-female ratio of 2.84:1, while Ng et al. (3) revealed that 61.7% of 128 patients were male, with a mean age of 78 years. These demographic patterns regarding age and gender are consistent with the findings presented in our case report.

The interpretation of MIC values for *S. indica*, as well as other *Shewanella* species, presents a considerable challenge due to the lack of species-specific clinical breakpoints in both CLSI and EUCAST guidelines. *Shewanella* can be classified as a non-*Enterobacterales* Gram-negative bacillus, which allows for MIC interpretation using CLSI breakpoints designated for ‘Other Non-*Enterobacterales*’ (CLSI M100, 34th edition, Table 2B–5, 2024). If a particular antimicrobial agent is not listed under “Other Non-*Enterobacterales*,” MIC interpretation is not possible using EUCAST either. In such cases, MIC values are reported without S/I/R categorization, accompanied by a note indicating the lack of species-specific breakpoints for *Shewanella* spp.

Significant differences in antimicrobial resistance among various *Shewanella* strains have been previously reported. Although it has been reported that *Shewanella* species can be resistant to ceftriaxone, susceptibility varies between strains, and our isolated *S. indica* was sensitive to ceftriaxone with MIC 4 μg/mL. Considering that the correlation between genotype and phenotype for aminoglycoside resistance was not established, we can assume that the aminoglycoside resistance genes were not expressed. In the present case report, *S. indica* exhibited a multidrug-resistant (MDR) phenotype. In contrast to the only available MIC data for *S. indica* published by Li et al. (17), which demonstrated susceptibility to the majority of the tested antibiotics, our findings revealed a distinctly different resistance profile. Furthermore, genome analysis of *S. indica* confirmed the presence of ARGs. Based on current research, *Shewanella* species may act as reservoirs of ARGs, which can be acquired or transmitted through horizontal gene transfer mechanisms such as transformation, conjugation, and transduction (1, 19). The presence of acquired ARGs and efflux pumps in the genome of *S. indica* supports the hypothesis that members of this genus may be emerging human pathogens capable of developing and disseminating resistance to multiple antibiotic classes. However, in this case report, we did not confirm the presence of plasmid sequences within the genome of the analyzed strain.

Accurate identification of *Shewanella* spp. remains challenging, as addressed in this study. We analyzed the presence of the *qnrA2* gene, which encodes a pentapeptide repeat protein that protects type II topoisomerases, thereby reducing susceptibility to quinolones and fluoroquinolones. Both MIC testing and genome analysis confirmed that the analyzed isolate was resistant to ciprofloxacin. The *qnrA2* gene was detected using ResFinder and BLAST analysis, supporting the classification of the isolate as *S. indica* rather than *S. algae,* in accordance with the findings of Araújo et al. (11). Furthermore, the absence of collagenase further supports the identification of the strain as *S. indica*, as described in the study of Tamez et al. (14).

Our *S. indica* strain also harbored multiple virulence-associated genes. Although the virulence phenotype was not experimentally assessed in this study, previous research has demonstrated the pathogenic potential of various *Shewanella* species (2). These bacteria possess a wide range of virulence factors, including flagella, type IV pili, quorum sensing systems, proteases, gelatinases, biofilm formation, and secretion systems, all of which contribute to host colonization and immune evasion (2, 14, 20, 21). The detection of similar virulence determinants in the *S. indica* genome in this case report suggests potential for pathogenicity and reinforces the need to consider *S. indica* as an emerging clinical threat.

Accurate identification of bacteria from the *Shewanella* genus remains a challenge in clinical microbiology due to their phenotypic similarity and the limitations of conventional diagnostic methods. This case highlights the shortcomings of identification using the VITEK 2 and MALDI-TOF MS systems, which were insufficient for precise species determination. At the same time, it emphasizes the importance of WGS as a reliable method for accurate identification and genomic characterization of unusual clinical isolates. This approach enables a deeper understanding of the pathogen’s genetic traits, which can have significant implications for diagnostics, treatment, and epidemiological surveillance. Additionally, the genome of *S. indica* presented in this case report has been deposited in the NCBI database and is publicly available for further analysis.

Regarding MALDI-TOF MS identification at the species level, our results are not in agreement with those reported by Yu et al. (25). They found that MALDI-TOF MS is a reliable and powerful tool for the rapid identification of *Shewanella* at the species level. The authors evaluated MALDI-TOF MS using 125 *Shewanella* test strains and compared it with the results of multilocus sequence analysis (MLSA). They found that 92.8% of the strains were correctly identified at the species level. The misidentified strains (*n* = 9) by MALDI-TOF MS involved five species of two groups, i.e., *Shewanella algae–Shewanella chilikensis–Shewanella indica* and *Shewanella seohaensis–Shewanella xiamenensis*. In order to overcome the limitations of MALDI-TOF MS, they used species-specific biomarker peaks that are discriminatory at the species level. With the use of the species biomarker peaks, nine misidentified test strains were accurately identified at the species level.

Although direct evidence of *S. indica* in the human gut remains limited, studies indicate that species within the *Shewanella* genus can function as opportunistic pathogens, particularly in individuals with various comorbidities, most notably hepatobiliary diseases and malignancies, and are frequently associated with polymicrobial infections (3). The three most common organisms found in co-infection with *Shewanella* were *Enterococcus*, *Escherichia coli*, and *Klebsiella* species (3).

Critically ill patients exhibit severe gut microbiota dysbiosis, characterized by reduced microbial diversity and expansion of opportunistic taxa, which can lead to the overgrowth of organisms with pathogenic potential (pathobionts) (22). In such conditions, organisms such as *Shewanella* spp. may exploit impaired gut barrier integrity and altered immune responses to colonize and persist. Evidence indicates that *Shewanella* species possess virulence factors such as biofilm-forming capacity, hemolysins, proteases, flagella, homoserine lactone signaling molecules, and gelatinases, which enable the invasion of human intestinal epithelial cells and enhance survival and pathogenicity in compromised hosts, representing key determinants in the initial stages of infectious disease (2, 23). Furthermore, *Shewanella* has been isolated from bile and drainage fluid in patients undergoing digestive tract surgeries, indicating its potential as an intestinal colonizer (24). These findings support the hypothesis that *S. indica*, such as other *Shewanella* species, could act as an opportunistic pathobiont in critically ill patients, particularly in complex clinical scenarios involving gut microbiome disruption.

In conclusion, the primary limitations of this retrospective case study are the difficulty in interpreting MIC values for *S. indica*, which reflects a broader challenge in evaluating antimicrobial susceptibility in *Shewanella* species. Additionally, *S. indica* was isolated only from the stool, and the presence of co-infections with other pathogens (e.g., MRSA, *C. difficile*) complicates the assessment of the direct clinical impact of *S. indica* in this case report. Nonetheless, the case has several strengths: (i) novelty: this is the first documented isolation of *S. indica* from a human clinical sample in Serbia and the broader Balkan region; (ii) genomic insight: WGS revealed the presence of ARGs and virulence-associated genes, expanding current knowledge of *S. indica*; (iii) clinical relevance: the case was analyzed in the context of a critically ill patient with multiple comorbidities, providing a realistic clinical scenario for emerging pathogens. An important takeaway lesson is the need to consider rare and emerging pathogens in critically ill patients, particularly when standard diagnostic tools yield inconclusive results.

## Data Availability

The datasets presented in this study can be found in online repositories. The names of the repository/repositories and accession number(s) can be found in the article/Supplementary material.

## Footnotes

1
https://blast.ncbi.nlm.nih.gov/Blast.cgi

2
https://cge.food.dtu.dk/services/PlasmidFinder/

3
https://blast.ncbi.nlm.nih.gov/Blast.cgi
