# Supplementary material for: First case report of Shewanella indica isolated from a hospitalized patient in Serbia
Source: Front Med (Lausanne). 2026 Jan 6;12:1715579. doi: 10.3389/fmed.2025.1715579 (PMC12816349; doi:10.3389/fmed.2025.1715579)
Supplement: Supplementary file 1 [file Table_1.docx]

| **Resistance gene** | **Identity** | **Aligment lengt/gene length** | **Position in reference** | **Contig or depth** | **Position in contig** | **Phenotype** | **PMID** | **Accession No.** |
| --- | --- | --- | --- | --- | --- | --- | --- | --- |
| aac(6')-Ib-Hangzhou | 99.81% | 519 / | 1...519 | NODE_14_length_119167_cov_149.73429 | 48908...49426 | ['tobramycin', 'amikacin'] | 19703723 | FJ503047 |
| blaOXA-SHE | 95.75% | 870 / | 1...870 | NODE_5_length_284755_cov_105.502080 | 92818...93687 | ['unknown beta-lactam'] | unpublished | AY066004 |
| blaOXA-2 | 99.64% | 828 / | 1...828 | NODE_14_length_119167_cov_149.734291 | 49514...50341 | ['amoxicillin', 'amoxicillin+clavulanic acid', 'ampicillin', 'ampicillin+clavulanic acid', 'ceftazidime', 'piperacillin'] | 2538329 | DQ112222 |
| mph(A) | 100.00% | 906 / | 1...906 | NODE_14_length_119167_cov_149.734291 | 41343...42248 | ['erythromycin', 'azithromycin', 'spiramycin', 'telithromycin'] | 8619599 | D16251 |
| qnrA2 | 100.00% | 657 / | 1...657 | NODE_1_length_643485_cov_107.473506 | 143203...143859 | ['ciprofloxacin'] | 18426894 | AY675584 |
| sul1 | 100.00% | 840 / | 1...840 | NODE_14_length_119167_cov_149.734291 | 47559...48398 | ['sulfamethoxazole'] | unpublished | U12338 |
| tet(A) | 100.00% | 1200 / | 1...1200 | NODE_14_length_119167_cov_149.734291 | 31229...32428 | ['tetracycline', 'doxycycline'] | 12654659 | AJ517790 |
