# Supplementary material for: First case report of Shewanella indica isolated from a hospitalized patient in Serbia
Source: Front Med (Lausanne). 2026 Jan 6;12:1715579. doi: 10.3389/fmed.2025.1715579 (PMC12816349; doi:10.3389/fmed.2025.1715579)
Supplement: Supplementary file 3 [file Table_3.docx]

Supplementary Table 3: MLST profile of *Shewanella indica*

Sequence type: Unknown

Nearest ST: 41

| Locus | Identity | Coverage | Alignment Length | Allele Length | Gaps | Allele |
| --- | --- | --- | --- | --- | --- | --- |
| 16S_rRNA | 100 | 85.29 | 1206 | 1414 | 0 | 16S_rRNA_15? |
| adk | 100 | 100 | 338 | 338 | 0 | adk_14 |
| atpB | 100 | 100 | 699 | 699 | 0 | atpB_4 |
| guaA | 99.0577 | 100 | 849 | 849 | 0 | guaA_23* |
| gyrB | 99.8405 | 100 | 627 | 627 | 0 | gyrB_4* |
| mdh | 100 | 100 | 381 | 381 | 0 | mdh_16 |
| recA | 99.8512 | 100 | 672 | 672 | 0 | recA_4* |
| rpoS | 100 | 100 | 481 | 481 | 0 | rpoS_2 |

Notes: *? alleles with less than 100% identity and 100% coverages found

* gyrB: Novel allele, ST may indicate nearest ST.

* guaA: Novel allele, ST may indicate nearest ST.

* recA: Novel allele, ST may indicate nearest ST.

? 16S_rRNA: Uncertain hit, ST can not be trusted.
